# Residual Black Wolfberry Fruit Improves Meat Quality of Sheep by Enhancing Immune and Antioxidant Capacity

**DOI:** 10.3390/vetsci12040324

**Published:** 2025-04-01

**Authors:** Pingping Duan, Yuxia Yang, Liangzhong Hou, Ying Wu, Jinlong Li, Congbin Xu, Tongjun Guo

**Affiliations:** 1 Feed Research Institute of Xinjiang Academy of Animal Husbandry Sciences, Urumqi 830011, China; 15770021628@163.com (P.D.); 15022947531@163.com (Y.Y.); xcb2318072452@163.com (C.X.); 2 Xinjiang Key Laboratory of Herbivorous Livestock Feed Biotechnology, Urumqi 830011, China; 3 Xinjiang Quality Basic Development Research Institute, Urumqi 830011, China

**Keywords:** residual black wolfberry fruit, sheep, serum, immunity, meat quality

## Abstract

The residual black wolfberry fruit (RBWF), rich in nutrients and active substances, has the potential to serve as an antibiotic alternative. This study evaluated the concentration-dependent effects of RBWF on serum biochemistry, immunity, antioxidant capacity, and meat quality of fattening sheep. Forty 3-month-old sheep were randomly assigned into four groups with 10 replicates in each group and fed experimental RBWF-supplemented diets (0%, 2%, 5%, and 8%). The results showed that RBWF supplementation significantly increased the serum levels of TP, BUN, ALT, AST, IgA, IgM, T-AOC, SOD, and GSH-Px in sheep (*p* < 0.05), while significantly decreasing the levels of LDH, TG, LDL-c, IgG, and MDA (*p* < 0.05). Furthermore, dietary RBWF significantly increased the VB1 and IMP levels in the longissimus dorsi muscle of sheep (*p* < 0.05) and significantly decreased the contents of fat and cholesterol (*p* < 0.05). Finally, RBWF increased the contents of C20:4 and Glu (*p* < 0.05). In conclusion, dietary supplementation with RBWF can improve the immune and antioxidant capacity of sheep and has a certain effect on improving the flavor of meat, of which 5% is the best.

## Simple Summary

*Lycium barbarum*, a renowned Chinese herbal medicine both on the national and international level, is abundant in polyphenols, polysaccharides, alkaloids, anthocyanins, and other bioactive components. These have a multitude of physiological effects, including the amelioration of immunity and anti-inflammatory properties. Moreover, considerable potential exists for the improvement of performance and quality of livestock products. For instance, studies have demonstrated that wolfberry extracts can improve growth performance, antioxidant capacity, and immunity, as well as increase the nutritional value of meat. *Lycium ruthenicum* (black wolfberry fruit) exhibits greater nutritional value and functionality than the *Lycium barbarum* (common wolfberry fruit), yet there is a paucity of studies examining its potential applications in ruminant animals. This study compared the effects of different proportions of residual black wolfberry fruit (RBWF) on serum biochemical, immune, and antioxidant levels as well as slaughter performance and meat quality of sheep. The results showed that adding 5% RBWF to the diet could improve the immunity of sheep and improve the flavor of meat. And 8% RBWF can improve the antioxidant capacity of sheep.

## 1. Introduction

The fruit, root, bark, and leaves of *Lycium barbarum* have medicinal value in traditional Chinese medicine [1]. The close relative *Lycium ruthenicum* Murr. contains a variety of nutrients and is rich in polyphenols, polysaccharides, alkaloids, anthocyanins, and other biochemical components that protect the organism against inflammation, oxidation, aging, tumor growth, and fatigue [2,3]. Chinese herbal medicine is rich in a variety of bioactive compounds that represent a good substitute for synthetic antibiotics [4]. In fact, previous studies indicated that—due to its variety in active substances—*Lycium barbarum* has multiple biological functions including antioxidant and immune regulation [5]. During the production process of fresh wolfberry, 5% to 10% of the residual fruit is generally discarded as waste. Considering that RBWF has a high nutritional value and contains a variety of bioactive substances—including *Lycium barbarum* polysaccharide (LBP) [6]—it is an easily available feed supplement.

Improved living standards and rapid development within animal husbandry has resulted in increased demands for high-quality livestock products. Agro-industrial by-products can be utilized in this direction. Particularly, according to the existing literature, dietary supplementation with Glycyrrhiza-derived polysaccharide extracts improved the serum biochemical characteristics and immune function of broilers [7]. Also, dietary supplementation with LBP significantly increased IgG, IgM, IL-10, IL-2, and TNF-α levels in weaned piglets [8]. Therefore, it is considered that RBWF might have great potential as a functional food to partially replace the conventional feed of ruminants.

Taking into consideration the nutritional characteristics of RBWF, it was incorporated into the sheep diet as a feed ingredient in the present experiment. Our previous studies [9] were primarily concentrated on the growth performance and rumen parameters of sheep. It was determined that the inclusion of 5% RBWF could enhance ADFI and ADG in sheep and concurrently improve rumen fermentation parameters in a dose-dependent manner. We speculate that the supplementation of RBWF in the diet of fattening sheep can maintain their health status by enhancing immunity. Thus, in order to provide a theoretical basis for the application of RBWF in sheep production, the current study aimed to analyze the concentration-dependent effects of RBWF on serum biochemical, immune, and antioxidant levels as well as slaughter performance and meat quality of sheep.

## 2. Materials and Methods

### 2.1. Ethics Committee Approval

The study was approved by the Science and Technology Ethics Committee of Xinjiang Academy of Animal Sciences, China (approval number 20230508). The procedures used adhered to the principles and regulations for ethical protection in human and animal biological science and technology in China.

### 2.2. Experimental Materials

**Figure 1:**
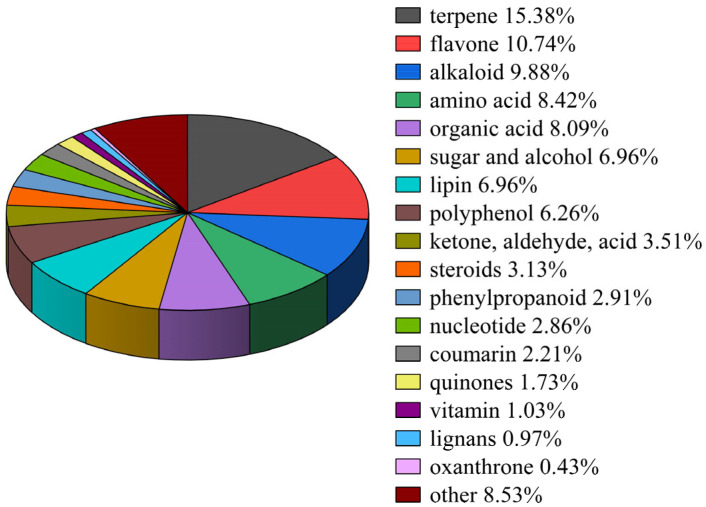
Classification of bioactive substances in RBWF.

In this experiment, following the collection of the black wolfberry fruit in October 2022, the fruit that did not meet the quality standard was identified as RBWF. The nutritional analysis of RBWF was then determined, as illustrated in Table 1. The nutritional components of RBWF were also assessed by liquid chromatography mass spectrometry (LC-MS), and 1853 active substances were detected, which fell into 18 categories (Figure 1).

### 2.3. Experimental Animals and Group Design

Following deworming, 40 male lambs (sheep, 4 months old, 29.85 ± 2.00 kg) were randomly assigned into 4 groups with 10 replicates per group through a single-factor experimental design based on body weight: control (CON group), experimental group I (RBWF2%), experimental group II (RBWF5%), and experimental group III (RBWF8%). The specific diet compositions and nutrient compositions are shown in Table 2.

The experiment lasted for 70 days, including a 10-day pre-experimental period and a 60-day experimental period.

### 2.4. Feeding Management

Prior to the experiment, the sheep house was thoroughly cleaned and disinfected while the sheep were subjected to a series of preparatory procedures including deworming and medicated bathing. During the experiment, the sheep were maintained in separate enclosures, and feed was provided twice daily at 10:00 and 18:00. Throughout the duration of the study, the sheep had free access to feed and water.

### 2.5. Sample Collection and Determination Index

#### 2.5.1. Serum Sample Collection

On Day 60 of the experiment, 5 mL blood was obtained twice from the jugular vein of each sheep and collected in 2 common vacuum blood collection tubes 2 h before the morning feeding (8:00). The tubes were left standing for 2 h and centrifuged at 3500 r/min for 15 min at 4 °C, and the resulting serum was stored at −80 °C until analysis.

The contents of total protein (TP), albumin (ALB), urea nitrogen (BUN), glucose (GLU), lactate dehydrogenase (LDH), creatine kinase (CK), triglyceride (TG), total cholesterol (TC), high density lipoprotein (HDL), low density lipoprotein (LDL), alanine aminotransferase (ALT), and aspartate aminotransferase (AST) in the serum were measured using a Mindray fully automatic biochemical analyzer (BS-480, Mindray Bio-Medical Electronics Co., Ltd., Shenzhen, China).

The levels of immunoglobulin A (IgA), immunoglobulin G (IgG), immunoglobulin M (IgM), superoxide dismutase (SOD), glutathione peroxidase (GSH-Px), malondialdehyde (MDA), and total antioxidant capacity (T-AOC) were determined by enzyme-linked immunosorbent assay (ELISA) (Shanghai Blue-based Biotechnology Co., Ltd., Shanghai, China) according to the manufacturer’s instructions and analyzed with a microplate reader (ELx800, BioTek, Winooski, VT, USA).

#### 2.5.2. Slaughter Performance

Before slaughter, the weight of each sheep was weighed and recorded as live weight (kg) before slaughter. The day after the end of the experiment, the sheep fasted for 12 h and deprived of water for 2 h, and then five sheep were selected from each group and stunned by electric shock according to the animal welfare protocol, followed by humane euthanasia by bleeding. The test sheep were bled through the carotid artery and jugular vein, and the head, hoof, skin, and viscera of the test sheep were removed, while the kidneys were retained. The hot carcass weight (HCW) was recorded. The longissimus dorsi muscle between the 12th and 13th ribs was cut, and the cross section of the eye muscle was printed with sulfuric acid paper. The longest (cm) and widest (cm) values were measured and recorded using a vernier caliper. The eye muscle area (cm^2^) was calculated as the longest value of the eye muscle cross section (cm) × the widest value (cm) × 0.7. The left longest dorsal (LD) muscle between the 7th and 12th ribs was isolated for the determination of amino acid and fatty acid composition and content. Backfat thickness was also measured and refers to the layer of fat positioned above the center of the eye muscle between the 12th and 13th ribs.

#### 2.5.3. Meat Quality

Meat quality was determined through the assessment of pH and chemical indicators including water, protein, fat, and ash content. The pH of carcass muscle was measured using a portable pH meter (Shanghai Instrument and Electrical Science Instrument Co., Ltd., Shanghai, China), which was calibrated with standard buffers (pH 4, 6.86, and 9.18). The moisture, crude protein, crude fat, and ash content of the meat were determined according to the AOAC method as described previously [10]. Thiamin (VB1), inosinic acid (IMP), and total cholesterol (TC) were determined by ELISA using an enzyme labeler (ELx800, BioTek, USA).

#### 2.5.4. Fatty Acid

The total fatty acids in frozen meat samples were extracted in accordance with the O Fallon (2007) method [11], and FAME separation was carried out by gas chromatography (GC-450, Varian Co., Walnut Creek, CA, USA) equipped with a special capillary column (Agilent J & W HP-88, 100 m × 0.25 mm × 0.2 μm) and a flame ionization detector (FID) (Agilent, Santa Clara, CA, USA). The flow rate of high-purity nitrogen was set at 25 mL/min while the FID utilized compressed air and high-purity hydrogen with optimal chromatography flow rates of 300 mL/min and 30 mL/min, respectively. The peak of the sample was determined according to the retention time. The concentration of a single fatty acid was determined according to the known standard (C4–C24 methyl ester mixture; Sigma-Aldrich, Inc., St. Louis, MO, USA).

#### 2.5.5. Amino Acid

To analyze the composition and concentration of amino acids, 100 mg of freeze-dried tissue sample was placed in 1.2 mL of 10% sulfosalicylic acid, shaken thoroughly, and centrifuged at 13,500× *g* for 15 min at 15 °C. Next, the resultant clear upper layer was filtered using a 0.22 μm membrane and transferred to a 2.0 mL glass screw vial, and the composition and concentration of amino acids in tissues were determined by a high-speed amino acid analyzer (L-8900, Hitachi High-Technologies Co., Tokyo, Japan).

### 2.6. Statistical Analysis

The data set was organized using Excel 2010, and a single-factor analysis of variance (ANOVA) was conducted with SPSS 23.0 for statistical analysis. The results from the tests were expressed as the mean and the standard error of the mean (SEM). A *p* < 0.05 was considered as a statistically significant difference while a 0.05 < *p* < 0.10 was considered as a trend. Statistical analysis was performed using bivariate correlation analysis in SPSS 26.0 statistical software, the Pearson algorithm was used to calculate the correlation coefficients, and heat maps of the correlation were created using the heat map tool in Hiplot Pro (https://hiplot.com.cn/) (accessed on 3 January 2025).

## 3. Results

### 3.1. Effects of Different RBWF Levels on the Growth Performance and Nutrient Apparent Digestibility of Sheep (n = 10)

Supplementary Table S1 showed growth performance and nutrient apparent digestibility, indicating that in our previous study [9], the RBWF5% group showed significantly higher ADFI, OM, and NDF apparent digestibility than the Control group (*p* < 0.05).

### 3.2. Effects of Different RBWF Levels on Serum Biochemistry of Sheep

The serum biochemical indexes of sheep are shown in Table 3. Compared to the Control group, serum levels of TP, ALB, BUN, ALT, and AST were significantly higher in the experimental groups. The increase in serum TP and ALB was quadratically affected with an increasing trend in RBWF2% and RBWF5% followed by a decrease in RBWF8% (Q, *p* < 0.05) whereas BUN showed an increase with increasing RBWF concentrations (*p* < 0.05). Compared to the Control group, serum levels of TG, LDL-c, and LDH were significantly decreased in the experimental groups (*p* < 0.05). The levels of TC, HDL-c, GLU, and CK were not significantly different between the experimental groups (*p* > 0.05).

### 3.3. Effects of Different RBWF Levels on Serum Immunity and Antioxidation in Sheep

The serum immune and antioxidant indexes of sheep are shown in Table 4. Compared to the Control group, RBWF resulted in significantly higher levels of IgA, IgM, T-AOC, SOD, and GSH-Px (*p* < 0.05) and significantly lower levels of IgG and MDA (*p* < 0.05) in all experimental groups.

### 3.4. Effects of Different RBWF Levels on Sheep Slaughter Performance

With increasing RBWF content in the diet, hot carcass weight and the eye muscle area of sheep showed a trend of increasing at first—showing the highest level in RBWF5%—followed by a decrease in RBWF8% (*p*-quadratic < 0.05) (Table 5). No significant differences were found between the Control group and the experimental groups for the other parameters analyzed (*p* > 0.05).

### 3.5. Effects of Different RBWF Levels on Physicochemical Characteristics in Sheep LD Muscle

Compared with the Control group, VB1 and IMP levels were significantly higher (*p* < 0.05) whereas fat and cholesterol levels were significantly lower (*p* < 0.05) in all experimental groups (Table 6). RBWF had no significant effect on moisture, protein levels, and ph (*p* > 0.05).

### 3.6. Effects of Different RBWF Levels on Amino Acid Composition in Sheep LD Muscle

The effect of RBWF on amino acid levels in sheep LD muscle is shown in Table 7. Twenty amino acids were detected in the muscle samples, including seven essential amino acids (EAAs). Compared with the Control group, the content of Glu was significantly increased in RBWF5% (*p* < 0.05).

### 3.7. Effects of Different RBWF Levels on Fatty Acid Composition in Sheep LD Muscle

The effect of RBWF on sheep LD muscle fatty acids is shown in Table 8. In total, 15 fatty acids were detected, including 8 saturated fatty acids and 7 unsaturated fatty acids. With increasing RBWF concentrations, C20:4 levels increased significantly (*p* < 0.05) in all experimental groups. RBWF had no significant effect on the content of other fatty acids (*p* > 0.05).

### 3.8. Correlation Analysis of Serum and Meat Quality

**Figure 2:**
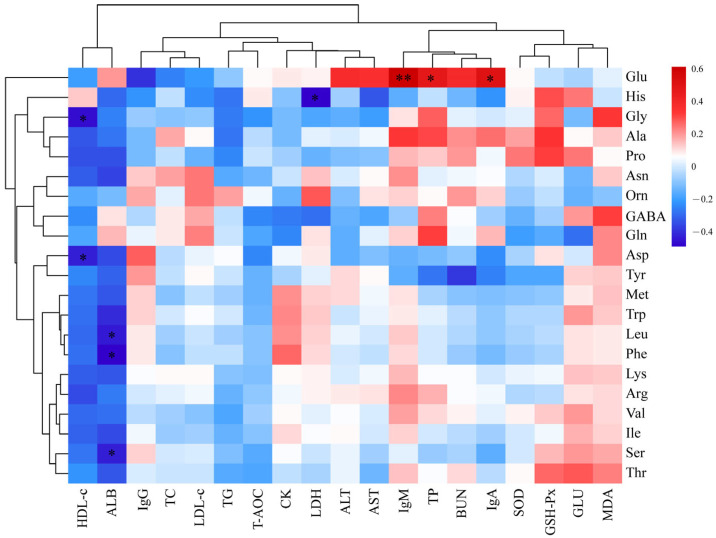
Pearson correlation and clustering analysis between serum indices and muscle amino acid content. Red color indicates a positive correlation, and blue color indicates a negative correlation. The color shade indicates the magnitude of the correlation. * represents 0.01 < *p* ≤ 0.05, and ** represents *p* ≤ 0.01.

**Figure 3:**
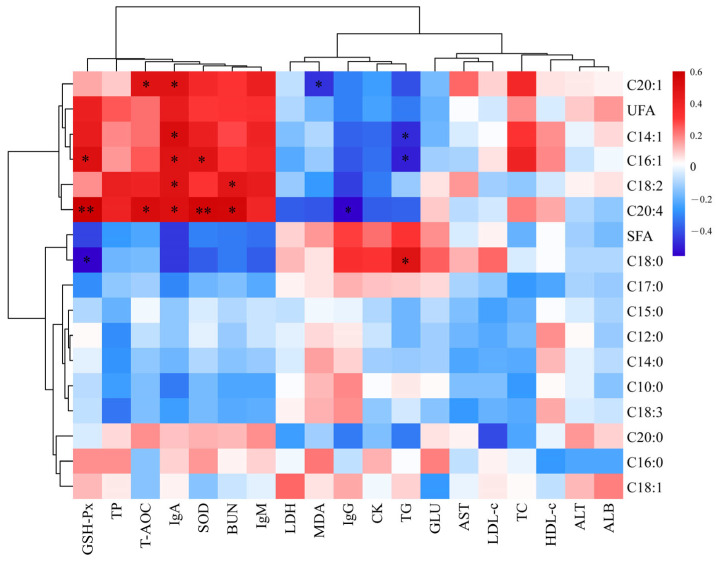
Pearson correlation and clustering analysis between serum indices and muscle fatty acid content. Red color indicates a positive correlation, and blue color indicates a negative correlation. The color shade indicates the magnitude of the correlation. * represents 0.01 < *p* ≤ 0.05, and ** represents *p* ≤ 0.01.

Heat map is an intuitive visualization method for cluster analysis of data and samples. The correlation of serum indexes with muscle amino acid or fatty acid content are shown in Figure 2 and Figure 3, respectively. Gly and Asp correlated negatively with HDL-c (*p* < 0.05). Ser, Leu, and Phe correlated negatively with ALB (*p* < 0.05). Glu correlated positively with TP, IgA, and IgM (*p* < 0.05). His correlated negatively with LDH (*p* < 0.05). C20:4 correlated positively with IgA, T-AOC, SOD, GSH-Px, and BUN and negatively with IgG (*p* < 0.05). C20:1 correlated positively with IgA and T-AOC and negatively with MDA (*p* < 0.05). C14:1 correlated positively with IgA and negatively with TG (*p* < 0.05). C16:1 correlated positively with GSH-Px, IgA, and SOD and negatively with TG (*p* < 0.05). C18:2 correlated positively with IgA and BUN and negatively with TG (*p* < 0.05). C18:0 correlated positively with TG and negatively with GSH-Px (*p* < 0.05).

## 4. Discussion

Apart from playing an important role in transporting nutrients to cells, blood is involved in regulation, protection, and homeostasis in mammals [12]. Consequently, the serum biochemical index is an important indicator reflecting the health status of animals. Not only does it reflect changes within organs and tissues as well as important metabolic characteristics, but it also indicates the physiological and nutritional status according to the internal and external environment of the animals [13,14]. For example, serum protein content is a parameter of nitrogen metabolism as well as an index for liver function and nutritional status. Also, high levels of TP and ALB in serum can promote the healthy growth of animals [15]. Finally, as a product of protein metabolism in the body, BUN content reflects the balance of protein catabolism and amino acids in the body and is closely related to the nitrogen content intake via the diet [16].

Previous studies indicated that addition of polysaccharide extracts to the diet can improve the serum biochemical characteristics and immune function of animals [17]. Our results show that increasing levels of dietary RBWF resulted in gradually increasing total protein, albumin, and urea nitrogen levels. These findings are consistent with the results of Li et al. [18] who reported that other Chinese herbal medicine additives increased the serum TP content in Longdong black goats, further suggesting that RBWF can promote protein digestion and absorption as well as protein utilization rate. As demonstrated in earlier research [9], the ADFI, apparent digestibility, of OM and NDF of RBWF5% was shown to be considerably elevated, indicating that RBWF may promote animal growth by synergistically regulating feeding behavior and nutrient utilization efficiency: on the one hand, it stimulates appetite to increase nutrient intake, and on the other hand, it improves feed conversion rate by improving the degradation ability of fiber substances. This could be due to the presence of active substances—such as polysaccharides—in RBWF that promote rumen fermentation, resulting in improved digestion and absorption of the nutrients in the diet, as such promoting healthy growth of sheep. Furthermore, this also indicates that RBWF can meet the growth needs of fattening sheep.

As the main source of energy, the dynamic balance of glucose absorption, transport, and metabolism is represented by the change of glucose content [19]. Changes in the activity of LDH—a NAD-dependent kinase that exists in a variety of tissues—can reflect tissue damage or disease status [20]. The main function of CK is promoting energy metabolism, especially during muscle contraction and ATP energy metabolism. Previous observations indicated that *Lycium barbarum* leaves improve blood glucose and blood lipid levels in diabetic rats by regulating metabolic processes and reversing intestinal flora imbalances [21]. In the current study, no significant differences in serum GLU and CK content was found. However, addition of RBWF to the diet could significantly reduce the serum LDH concentration of sheep. It is hypothesized that RBWF may reduce the damage of oxidative stress to cells by neutralizing free radicals through its antioxidant components (*Lycium barbarum* polysaccharides and flavonoids), thereby inhibiting enzyme leakage caused by exercise or metabolic stress. TC and TG contents mainly reflect the fat deposition and lipid metabolism in the body. In fact, TG is the most abundant lipid in animals and functions as energy supply and energy storage. Also, whereas TC—with its important components HDL-c and LDL-c—exists in all tissues of animals, its presence in blood mainly derives from exogenous absorption as well as endogenous synthesis. Previous studies indicated that LBP limits fat formation through a dose-dependent reduction of lipid accumulation and downregulation of key transcription factors and proteins involved in the fat-formation pathway [22]. Also, Zhuang et al. demonstrated that LBP reduced TC and LDL-c levels in fish while promoting fatty acid oxidation and hydrolysis of triglycerides [23]. Moreover, Li et al. provided evidence that dietary supplementation with Chinese herbal medicine mixtures reduced the serum levels of cholesterol and triglycerides in laying hens [24]. In the current study, addition of RBWF to the diet significantly reduced serum TG and LDL-c levels of sheep. This may be due to the fact that some components of RBWF, such as *Lycium barbarum* polysaccharides and phytosterol, have the effect of regulating blood lipids, which can interfere with the absorption and metabolism of cholesterol and promote the excretion of cholesterol, thereby reducing the concentration of TG and LDL-c in serum [25]. It is evident that further study is required in order to fully comprehend the regulatory mechanism of RBWF.

Immunoglobulins are proteins with non-specific immunity that function to isolate pathogens. The content of immunoglobulins in serum is an important index representing body immune function [26]. Zhang et al. evidenced that addition of fermented wolfberry residue to the diet of sheep significantly increased the levels of IgG, IgA, IgM, and GLB while inducing expression of immune-related pathway genes resulting in improved immunity [27]. Consistent with previous findings of Ju et al. [28], the current study indicated that RBWF significantly increased serum levels of IgA and IgM in sheep. This suggests that RBWF could promote the function of immune cells thereby improving the immunity of livestock and poultry. The specific mechanism through which RBWF promotes immunity has not been studied. It is hypothesized that some metabolites of the RBWF-derived active ingredients could be absorbed by the rumen and intestinal flora and participate in the immune process and enhancing the immunity of the body. During growth, animals encounter a variety of stimuli that affect the body’s redox balance, resulting in increased MDA levels in the body and a decline in animal production performance [29]. Supplementation with exogenous antioxidants can improve the body’s antioxidant status [30]. For example, polysaccharides regulate the expression of downstream antioxidant enzymes through the endogenous antioxidant stress Nrf2/ARE pathway, resulting in significantly improved antioxidant capacity and reduced oxidative stress-related damage [31]. In fact, LBP promotes the repair and regeneration of cavernous nerve injury by increasing serum SOD and GSH-Px activity and decreasing MDA activity [32]. Also, previous studies evidenced that LBP resulted in upregulated expression of genes involved in fatty acid oxidation in the liver, resulting in reduced serum TC and TG levels in serum and reduced TG levels in the liver [33]. Long et al. reported that dietary supplementation with 2000 mg/kg LBP improved the growth performance, digestive enzyme activity, antioxidant capacity, and immune function of broilers [34]. Consistent with previous studies, the current study showed that dietary supplementation with RBWF resulted in significantly increased levels of T-AOC, SOD, and GSH-Px and reduced levels of MDA in sheep serum. It is hypothesized that active substances such as LBP and betaine contained in RBWF might enhance the body’s antioxidant capacity by mediating the humoral immunity and regulating the body’s physiological function. This should be addressed in future studies. Guo et al. previously reported that *Lycium barbarum* leaves had no significant effect on the main antioxidant enzymes in rats [35]. This inconsistent observation could be due to differences in animal species and/or feeding concentrations. As demonstrated in previous studies [9], the simultaneous optimization of nutrient intake and utilization efficiency is directly responsible for enhancing growth performance. However, this effect is not isolated, as evidenced by serum immune and antioxidant index analysis, which revealed a significant increase in the concentrations of IgA, IgM, T-AOC, SOD and GSH-Px in RBWF5%. This suggests that it may indirectly promote growth through the ’antioxidant–immune–digestive’ interaction mechanism. In the future, it is necessary to analyze the dynamic regulation of the ’digestion–immunity–growth’ ternary relationship under nutritional intervention through joint research, so as to provide a theoretical foundation for the design of precise nutritional strategies.

Yu et al. previously reported a significant correlation between serum biochemical indexes, slaughter traits, and meat quality [36]. Also, Tao et al. reported improved slaughter performance of finishing pigs following dietary supplementation with LBP due to significant increases in final weight, slaughter rate, and carcass length as well as reduced feed-to-weight ratio, backfat thickness, shear force, and cooking loss rate [37]. In addition, increased final weight and eye muscle area as well as improved meat quality indicators were reported in Hu sheep following dietary supplementation with *Lycium barbarum* branches and leaves [38]. In the current experiment, RBWF had no significant effect on the slaughter traits of sheep. However, a secondary effect on the carcass weight and eye muscle area of sheep was observed, showing a trend of concentration-dependent increase with RBWF concentrations up to 5% and subsequent decrease with a RBWF concentration of 8%. Previous reports indicated that IMP can enhance the umami of meat resulting in a more delicious taste [39]. In the current study, no significant effect on moisture, protein, and pH of longissimus dorsi muscle was found. However, a significantly increased VB1 and IMP contents were observed, suggesting that RBWF could increase the umami taste of mutton.

Amino acids are the basic unit of muscle protein, and their content directly affects the nutritional value of meat [40]. Previous studies evidenced that the closer the proportion of essential amino acids in meat to human needs, the higher its nutritional value [41]. For example, goat meat is a natural green, nutritious, and healthy food with a complete variety and rich content of amino acids [42]. Amino acids also play a key role in the formation of meat flavor. Glu is one of the most important umami amino acids in meat with a high impact on meat flavor [43]. In addition, together with other amino acids, Glu also affects the tenderness and juiciness of meat [44]. The muscular amino acid content and composition are affected by feeding conditions and feed ingredients. For example, dietary supplementation with specific amino acids improves the intramuscular fat content and fatty acid composition of the meat with a high impact on overall meat quality [45]. In the current experiment, dietary supplementation with RBWF significantly increased the Glu content in meat but not that of other amino acids. Next to being an important source of energy supply, fatty acids also participate in cell signal transduction and gene-expression regulation with regulatory functions in multiple cell types [46]. Differences in fatty acid composition have a significant effect on meat quality [47]. For example, pork and chicken contain a higher proportion of PUFA, especially n-6 fatty acid linoleic acid (LA) [48], while beef and mutton have a high ratio of SFA to PUFA [49,50]. These differences affect the texture, flavor, and oxidative stability of meat [51]. In particular, n-3 PUFA—such as α-linolenic acid (ALA)—has a beneficial effect on cardiovascular health and is high in beef and mutton [52]. In addition, the ω-6 PUFA C18:2 contributes to prevention of cardiovascular disease by regulating lipoprotein cholesterol levels in the blood [53]. Previous studies evidenced improved nutritional and health values of meat through changing the feed structure and nutrients of ruminants [54]. However, this could also lead to oxidation problems during meat processing, which could affect its flavor and quality [55]. Saturated fatty acids (SFAs) have a negative impact on musculoskeletal tissue, mainly because palmitic acid (PA, 16:0) can reduce collagen content [56]. Unsaturated fatty acids have many positive effects on human health and are indispensable nutrients for maintaining physiological functions and promoting health [57]. The oxidation of unsaturated fatty acids will lead to a decline in meat quality and affect the shelf life of meat. At the same time, the oxidation products of fatty acids, such as volatile substances produced during cooking, have an important impact on the flavor of meat [58]. The current study showed that dietary supplementation with RBWF increased the muscular C20:4 contents, with no significant effect on other fatty acids. Overall, the current data evidences that RBWF resulted in improved meat flavor with no adverse effect on meat quality. Combined with serum data analysis indicating RBWF’s capacity to increase the body’s immune level, these observations further suggest that RBWF might be beneficial in cardiovascular disease prevention.

Finally, our study revealed correlations between multiple serum indicators and the muscular amino acid and fatty acid content. For example, Glu s correlated positively and significantly with serum TP, IgA, and IgM levels whereas TG and HDL were correlated negatively with most amino acids. These observations suggest that dietary RBWF could result in increased serum TP content and immunity in sheep, promote lipid metabolism, increase Glu content in muscle, and improve muscle flavor. Previous studies reported that C20:4 is a precursor of a variety of bioactive substances, such as prostaglandins, leukotrienes, and thromboxane. These substances play an important role in regulating inflammation, vasoconstriction, and platelet aggregation [59]. The current study reported a positive correlation of C20:4 with T-AOC, SOD, and GSH-Px. Thus, dietary supplementation of RBWF could significantly increase the serum antioxidant capacity of sheep and increase the content of C20:4. However, the specific mechanism of RBWF on serum indicators and meat quality still needs further study.

## 5. Conclusions

The results of the current study evidenced that dietary supplementation of animal feed with RBWF improves serum immunity and antioxidant capacity of sheep. Dietary supplementation with RBWF significantly increased muscular VB1 and IMP levels and increased its C20:4, and Glu content resulted in enhanced meat quality and flavor. Overall, the recommended dietary supplement for RBWF is 5%. However, the mechanism of the effect of RBWF supplementation on the physiological level and meat quality of sheep needs further study. RBWF has been demonstrated to provide multi-target support for the improvement of growth performance of sheep by means of synergistic regulation of antioxidant defense, immune homeostasis, and efficient fiber utilization. However, its dose-dependent effect highlights the complex interaction of ’active ingredient-microbial metabolism-host response’ in ruminant nutrition intervention. In the future, it is necessary to analyze the dynamic balance mechanism of RBWF active substances in ’redox–immunity–growth’ through multi-omics joint analysis, so as to provide a theoretical tool for precision animal husbandry.

## Figures and Tables

**Table 1:** The main nutritional components of RBWF (DM basis, %).

Items	Content
DM	93.90
CP	14.29
EE	11.46
Ash	9.20
CF	8.86
ADF	16.60
NDF	28.00
Ca	0.24
Pi	0.30

**Table 2:** Composition and nutrient levels of the basal diet (DM basis, %).

Items	Group			
	Control Group	RBWF2%	RBWF5%	RBWF8%
Ingredients (%)				
Corn	33.80	35.00	32.50	31.00
Wheat bran	9.00	6.40	6.28	5.15
Cottonseed meal	13.70	13.30	12.92	12.65
RBWF	0.00	2.00	5.00	8.00
Corn stalk	18.00	18.00	18.00	17.90
Alfalfa	20.50	20.30	20.30	20.30
Premix ^1^	5.00	5.00	5.00	5.00
Nutrient components ^2^				
DM (%)	92.64	92.66	92.67	92.67
ME (MJ/kg)	11.06	11.02	10.98	10.93
CP (%)	13.96	13.97	13.99	13.99
NDF (%)	31.12	31.14	31.24	31.45
ADF (%)	16.80	16.85	17.07	17.30
Ca (%)	0.70	0.93	1.16	1.10
P (%)	0.49	0.56	0.64	0.41

^1^ The premix provided the following per kg of the diet: VA 150,000 IU, VD3 56,500 IU, VE 8000 IU, Se (as sodium selenite) 14 mg, I (as potassium iodide) 58 mg, Cu (as copper sulfate) 290 mg, Mn (as manganese sulfate) 1925 mg, Zn (as zinc oxide) 2050 mg, and Co (as cobalt sulfate) 24 mg. ^2^ Calculated values.

**Table 3:** Effects of different RBWF levels on serum biochemistry of sheep (n = 10).

Items	Control Group	RBWF2%	RBWF5%	RBWF8%	SEM	*p*-Value	
						Trt	Q
TP (g/L)	60.13 ^c^	70.61 ^ab^	75.30 ^a^	67.40 ^b^	1.302	<0.001	<0.001
ALB (g/L)	27.46 ^b^	29.73 ^a^	29.50 ^a^	27.16 ^b^	0.638	0.013	0.001
BUN (mmol/L)	5.21 ^c^	6.78 ^b^	7.83 ^a^	8.22 ^a^	0.216	<0.001	0.061
TG (mmol/L)	0.39 ^a^	0.24 ^b^	0.26 ^b^	0.17 ^c^	0.017	<0.001	0.182
TC (mmol/L)	1.51	1.52	1.52	1.58	0.042	0.938	0.752
HDL-c (mmol/L)	0.53	0.50	0.46	0.51	0.013	0.249	0.125
LDL-c (mmol/L)	0.78 ^a^	0.65 ^b^	0.63 ^b^	0.55^c^	0.032	0.030	0.654
GLU (mmol/L)	4.35	4.56	4.31	4.81	0.072	0.052	0.320
LDH (U/L)	177.85 ^a^	132.02 ^c^	165.49 ^b^	115.81 ^c^	5.480	<0.001	0.807
CK (U/mL)	0.23	0.18	0.17	0.14	0.013	0.154	0.620
ALT (U/L)	6.47 ^c^	7.03 ^b^	8.90 ^a^	7.14 ^b^	0.369	0.034	0.110
AST (U/L)	19.21 ^b^	24.76 ^ab^	28.16 ^a^	24.94 ^ab^	1.145	0.041	0.047

^a,b,c^ Different superscripts indicate significant differences within a row (*p* < 0.05). SEM is the pooled standard error between five groups; the *p*-value indicates significance. Trt, treatment effect; L, linear; Q, quadratic.

**Table 4:** Effects of different RBWF levels on immunity and antioxidant parameters in serum of in sheep (n = 10).

Items	Control Group	RBWF2%	RBWF5%	RBWF8%	SEM	*p*-Value	
						Trt	Q
IgA (g/L)	0.51 ^c^	1.66 ^b^	2.32 ^a^	1.81 ^b^	0.12	<0.001	<0.001
IgG (g/L)	20.60 ^a^	10.26 ^b^	10.18 ^b^	8.47 ^b^	0.904	<0.001	<0.001
IgM (g/L)	1.11 ^c^	2.25 ^b^	2.91 ^a^	2.26 ^b^	0.119	<0.001	<0.001
T-AOC (U/mL)	6.52 ^d^	6.99 ^c^	7.36 ^b^	7.76 ^a^	0.091	<0.001	0.766
SOD (U/mL)	53.93 ^d^	60.95 ^c^	67.94 ^b^	79.44 ^a^	1.561	<0.001	0.011
GSH-Px (U/mL)	123.38 ^d^	131.04 ^c^	142.40 ^b^	158.52 ^a^	2.69	<0.001	0.229
MDA (nmol/mL)	4.98 ^a^	4.65 ^a^	4.39 ^ab^	3.96 ^b^	0.107	0.003	0.792

^a,b,c,d^ Different superscripts indicate significant differences within a row (*p* < 0.05). SEM is the pooled standard error between five groups; the *p*-value indicates significance. Trt, treatment effect; Q, quadratic.

**Table 5:** Effects of different RBWF levels on sheep slaughter performance (n = 5).

Items	Control Group	RBWF2%	RBWF5%	RBWF8%	SEM	*p*-Value	
						Trt	Q
Live weight before slaughter (kg)	43.77	43.09	45.06	42.70	0.527	0.522	0.076
Hot carcass weight (kg)	21.90	21.20	22.68	20.74	0.269	0.202	0.047
Eye muscle area (cm^2^)	12.34	14.58	14.25	13.81	0.463	0.314	0.039
Backfat thickness (mm)	4.33	4.61	4.56	4.42	0.053	0.510	0.128

Trt, treatment effect; Q, quadratic.

**Table 6:** Effects of different RBWF levels on physicochemical characteristics in sheep LD muscle (n = 5).

Items	Control Group	RBWF2%	RBWF5%	RBWF8%	SEM	*p*-Value	
						Trt	Q
ph_45min_	6.89	6.92	6.76	6.62	0.061	0.346	0.512
Protein (%)	19.89	21.35	21.99	22.22	1.233	0.143	0.453
Ether extract (%)	6.36 ^a^	5.90 ^a^	5.81 ^ab^	5.29 ^b^	0.349	<0.001	0.007
Ash (%)	4.69	4.67	4.71	4.64	0.012	0.331	0.109
Moisture (%)	74.14	71.87	72.79	73.41	0.297	0.072	0.028
VB1	0.38 ^c^	0.75 ^b^	0.90 ^b^	1.08 ^a^	0.072	<0.001	0.039
IMP	30.82 ^b^	328.74 ^a^	121.39 ^ab^	223.76 ^ab^	48.115	0.156	0.349
CHO	53.13 ^a^	38.66 ^b^	24.95 ^c^	23.70 ^c^	3.081	<0.001	0.063

^a,b,c^ Different superscripts indicate significant differences within a row (*p* < 0.05). SEM is the pooled standard error between five groups; the *p*-value indicates significance. Trt, treatment effect; Q, quadratic.

**Table 7:** Effects of different RBWF levels on amino acid composition in sheep LD muscle (n = 5).

Items	Control Group	RBWF2%	RBWF5%	RBWF8%	SEM	*p*-Value	
						Trt	Q
Gly	67.56	74.13	75.98	70.61	3.141	0.815	0.376
Ala	279.98	283.79	316.66	310.93	11.165	0.589	0.796
GABA	3.85	5.56	3.52	4.04	0.337	0.159	0.454
Ser	85.58	67.41	87.27	79.87	5.715	0.656	0.727
Pro	35.28	35.08	39.94	41.50	2.368	0.732	0.885
Val	55.56	49.77	66.35	56.68	4.501	0.652	0.765
Thr	44.98	40.24	49.36	45.83	3.040	0.797	0.985
Ile	44.43	37.41	49.93	41.45	3.220	0.597	0.827
Leu	102.15	82.32	115.50	90.61	7.090	0.391	0.751
Asn	53.19	51.83	55.65	51.13	2.908	0.953	0.773
Orn	18.50	16.54	18.17	16.80	1.829	0.980	0.965
Asp	37.02	23.75	29.18	30.38	3.400	0.656	0.345
Gln	296.10	408.86	297.14	288.29	21.330	0.158	0.188
Lys	87.98	74.99	97.94	85.74	7.948	0.815	0.958
Glu	78.80 ^c^	94.09 ^ab^	104.22 ^a^	84.99 ^c^	5.261	0.006	0.002
Met	39.83	33.58	43.23	33.96	2.513	0.468	0.677
His	111.23	116.77	117.81	124.49	8.044	0.959	0.965
Phe	54.83	44.16	61.89	48.26	3.560	0.320	0.718
Arg	75.31	75.17	86.95	72.81	4.735	0.723	0.446
Tyr	53.22	45.61	47.36	40.85	4.250	0.809	0.980
Trp	12.93	9.97	13.72	11.56	0.951	0.564	0.932

^a,b,c^ Different superscripts indicate significant differences within a row (*p* < 0.05). SEM is the pooled standard error between five groups; the *p*-value indicates significance. Trt, treatment effect; Q, quadratic.

**Table 8:** Effects of different RBWF levels on fatty acid composition in sheep LD muscle (n = 5).

Items	Control Group	RBWF2%	RBWF5%	RBWF8%	SEM	*p*-Value	
						Trt	Q
SFA	55.70	53.49	53.10	52.20	0.737	0.409	0.662
UFA	44.30	46.51	46.90	47.80	0.737	0.409	0.662
C10:0	0.28	0.22	0.24	0.19	0.027	0.714	0.962
C12:0	0.34	0.31	0.31	0.26	0.055	0.969	0.945
C14:0	4.25	4.00	3.83	3.45	0.356	0.900	0.933
C14:1	0.11	0.27	0.26	0.38	0.047	0.246	0.809
C15:0	0.48	0.49	0.48	0.42	0.044	0.947	0.706
C16:0	26.81	26.63	27.03	27.11	0.164	0.759	0.721
C16:1	1.48	2.23	2.20	3.03	0.236	0.135	0.921
C17:0	1.32	1.27	1.25	1.13	0.060	0.738	0.784
C18:0	22.11	20.27	19.47	19.33	0.606	0.365	0.488
C18:1	37.65	38.39	38.14	36.93	0.708	0.989	0.988
C18:2	2.93	3.28	4.08	3.51	0.163	0.076	0.131
C20:0	0.12	0.15	0.15	0.13	0.010	0.637	0.214
C20:1	0.06	0.09	0.10	0.13	0.011	0.237	0.995
C18:3	0.71	0.50	0.50	0.36	0.109	0.755	0.870
C20:4	0.12 ^b^	0.17 ^ab^	0.21 ^a^	0.23 ^a^	0.014	0.040	0.628

^a,b^ Different superscripts indicate significant differences within a row (*p* < 0.05). SEM is the pooled standard error between five groups; the *p*-value indicates significance. Trt, treatment effect; Q, quadratic.

## Data Availability

The original contributions presented in this study are included in the article/Supplementary Material. Further inquiries can be directed to the corresponding author(s).

## Supplementary Materials

The following supporting information can be downloaded at: https://www.mdpi.com/article/10.3390/vetsci12040324/s1, Table S1: Effects of RBWF on growth performance and apparent digestibility of nutrients of sheep (n = 10).
