# Supplementary material for: Residual Black Wolfberry Fruit Improves Meat Quality of Sheep by Enhancing Immune and Antioxidant Capacity
Source: Vet Sci. 2025 Apr 1;12(4):324. doi: 10.3390/vetsci12040324 (PMC12030814; doi:10.3390/vetsci12040324)
Supplement: Supplementary file 1 [file vetsci-12-00324-s001.zip › vetsci-3521500-supplementary.pdf]

Supplementary Table S1. Effects of RBWF on growth performance and apparent digestibility of nutrients of sheep (n=10).

Items[1]	Control group	RBWF2%	RBWF5%	RBWF8%	SEM	*P*-value[2]	
						Trt	Q
Growth performance							
Initial body weights, kg	29.91	30.05	29.90	29.54	0.230	0.901	0.605
Final weight, kg	43.77	43.09	45.06	42.70	0.557	0.522	0.526
ADG, g	249.29	231.19	261.18	230.48	8.394	0.563	0.788
total gain weight, kg	14.21	13.18	14.89	13.14	0.478	0.563	0.788
ADFI, kg	1.79^{b}	1.73^{b}	1.84^{a}	1.65^{c}	0.043	0.021	<0.001
F:G	7.52	7.78	7.23	7.67	0.271	0.925	0.909
Digestibility							
DM	70.60	68.80	71.20	66.90	0.638	0.059	0.277
OM	59.76^{b}	58.64^{b}	61.61^{a}	56.46^{c}	0.577	0.047	0.144
CP	73.26	73.04	73.45	72.63	0.173	0.397	0.396
EE	55.67	56.24	56.43	56.57	0.418	0.896	0.809
NDF	43.72^{b}	44.76^{ab}	47.55^{a}	41.81^{b}	0.677	0.010	0.005
ADF	24.93	25.88	27.74	23.69	0.634	0.135	0.048

[1] ADG, average daily gain; ADFI, average daily feed intake; F:G, ADFI/ADG.

[2] Trt = treatment effect, Q = quadratic.

[a,b] Different superscripts indicate significant differences within a row ($P < 0.05$). SEM is the pooled standard error between five groups; the *P*-value indicates significance.
